# White light-emitting electrochemical cells based on metal-free TADF emitters

**DOI:** 10.1038/s41467-025-55954-3

**Published:** 2025-01-14

**Authors:** Shi Tang, Youichi Tsuchiya, Jia Wang, Chihaya Adachi, Ludvig Edman

**Affiliations:** 1 The Organic Photonics and Electronics Group, Department of Physics, Umeå University, Umeå, Sweden; 2 LunaLEC AB, Umeå, Sweden; 3 Center for Organic Photonics and Electronics Research (OPERA), Kyushu University, Fukuoka, Japan; 4 Wallenberg Initiative Materials Science for Sustainability, Department of Physics, Umeå University, Umeå, Sweden

**Keywords:** Organic LEDs, Electronic devices, Electrical and electronic engineering

## Abstract

The attainment of white emission from a light-emitting electrochemical cell (LEC) is important, since it enables illumination and facile color conversion from devices that can be cost-efficient and sustainable. However, a drawback with current white LECs is that they either employ non-sustainable metals as an emitter constituent or are intrinsically efficiency limited by that the emitter only converts singlet excitons to photons. Organic compounds that emit by thermally activated delayed fluorescence (TADF) can address these issues since they can harvest all excitons for light emission while being metal free. Here, we report on the first white LEC based on solely metal-free TADF emitters, as accomplished through careful tuning of the energy-transfer processes and the electrochemically formed doping structure in the single-layer active material. The designed TADF-LEC emits angle-invariant white light (color rendering index = 88) with an external quantum efficiency of 2.1 % at a luminance of 350 cd/m^2^.

## Abstract (web summary)

Current white light-emitting electrochemical cells either consist of non-sustainable metal emitters or are intrinsically efficiency limited. Here, metal-free thermally activated delayed fluorescent emitters are employed and tuned for the achievement of broadband white light emission.

## Introduction

Electroluminescent (EL) technologies that efficiently deliver broadband white emission from conformable, lightweight, and non-glaring large-area surfaces are projected to pave the way for a paradigm shift in illumination and also enable a wide range of emissive applications in healthcare^1^, displays^2^, security^3^, and communication^4^. The point-like light-emitting diode (LED) is the most efficient and long-lasting EL technology as of today^5^, while the organic LED (OLED) instead offers large-area emission from lightweight and flexible device architectures^6,7^. However, from a sustainability viewpoint, it is a drawback that current commercial LED and OLED devices are fabricated by energy-intensive thermal evaporation under vacuum, and that they comprise geopolitically problematic materials, such as iridium, platinum, and rare earths, which are cumbersome to mine and recycle^8–10^.

The light-emitting electrochemical cell (LEC) is a less investigated and developed EL technology that is similar in appearance to the OLED, but which is formally distinguished by the existence of mobile ions in its active material^11^. These mobile ions redistribute during the initial LEC operation for the formation of electric double layers (EDLs) at the electrode interfaces and for the in-situ establishment of a p-n junction doping structure by electrochemical doping. These two mobile-ion-induced events enable efficient injection, transport, and recombination of holes and electrons within a very robust and simple LEC device structure, which can comprise two air-stabile electrodes sandwiching a single-layer active material. This air-stabile device structure has in turn paved the way for energy- and cost-efficient fabrication of complete LEC devices by scalable solution-based printing and coating under ambient air^12–17^.

The original report on white emission from a LEC device was by Yang and Pei in 1997, who employed a fluorescent conjugated polymer (CP) as the single emitter^18^. The authors suggested that the origin of the broadband EL was significant phase separation between the CP emitter and the poly(ethylene oxide)-rich electrolyte without going into further detail^18^. Su and Peng and co-workers were first to utilize a phosphorescent compound for the attainment of white emission from an LEC in 2008 when they combined blue-green and red emitting iridium complexes in the active material; the resulting phosphorescent LEC delivered white emission with a color rendering index (CRI) of 80, at an external quantum efficiency (EQE) of 3.3% and a luminance of 43 cd/m^2^ ^19^. In 2014, Watkins et al. introduced quantum dots (QDs) as the emissive species in an LEC, and by tuning the ratio between blue-, green- and red-emitting CdSe/CdS QDs they realized a LEC that emitted white EL with CIE coordinates of (0.33, 0.33)^20^.

Following these pioneering demonstrations, a significant number of white-emitting LEC devices has been reported in the scientific literature, with the emitters being fluorescent CPs^21–24^, fluorescent small molecules^25,26^, phosphorescent transition metal complexes (TMC)^27–36^, QDs^37^, or a combination thereof^38–41^. Table 1 presents a summary of the key published performance metrics of white LECs, which were reported to emit with a luminance exceeding 100 cd/m^2^. We note that for the voltage-driven devices, the presented efficiency (EQE or current efficacy) is commonly not measured at the presented peak luminance. We have selected to organize these white LECs into two categories based on the properties of the emissive species: (i) “metal-based emitters that can harvest both singlets and triplets for emission” and (ii) “metal-free emitters that only harvest singlets for emission”. We call attention to that all reported white-emitting LECs to date are limited in that they either employ rare metals for the emitter or only can convert the electrically generated singlets to photons. This fact poses an obvious drawback, not the least from a sustainability perspective.

**Table 1:** Performance of white LECs with a reported peak luminescence exceeding 100 cd/m^2^, as organized by emitter type

Emitter	CIE coordinates (*x*, *y*)	CRI^b^	Peak luminance (cd/m^2^)^c^	Current efficacy (cd/A)^c^	EQE (%)^c^	Lifetime at >100 cd/m^2^ (h)	Year^ref^	Driving protocol
Metal-based emitters that harvest both singlets and triplets for emission								
Ir-TMC	0.45, 0.44	81	106	9.8	4.7	≈3.3	2009^27^	Potentiostatic (4 V)
Ir-TMC	0.38, 0.47		845	8.5		>0.25	2014^66^	Galvanostatic (10 mA/cm^2^)
CdSe/CdS QDs	0.33, 0.33		≈100^b^	0.39			2014^20^	Potentiostatic (10 V)
Ir-TMC + CP	0.37, 0.45	79	250	4.7			2015^39^	Galvanostatic (5.8 mA/cm^2^)
x-CP^a^	0.35, 0.37		1241	8.1		>13.3^b^	2015^38^	Galvanostatic (13.3 mA/cm^2^)
Ir-TMC + CP	0.28, 0.31	96	191		0.076	≈0.004^b^	2017^41^	Potentiostatic (4 V)
TADF molecule + Ir-TMC	0.39, 0.37	61	1220	1.8			2017^52^	Voltage scan (peak luminance at 14 V)^b^
Ir-TMC	0.42, 0.41		1032	10.1			2017^67^	Voltage scan (peak luminance at 7 V)
Ir-TMC	0.45, 0.36	68	1105		0.53	>0.03^b^	2018^68^	Pulsed voltage (*V*_max_ = 10 V)
CdSe/ZnS QD + Ir-TMC		80	873	0.46	0.23		2018^69^	Voltage scan (peak luminance at 11 V)
Ir-TMC	0.39, 0.39	79	3956	17.4	8.9	≈3.3^b^	2020^28^	Voltage scan (peak luminance at 7.5 V)^b^
Metal-free emitters that only harvest singlets for emission								
CP			400		2.4		1997^18^	Voltage scan (peak luminance at 4 V)
CP	0.24, 0.31		257		0.15		2010^70^	Voltage scan (peak luminance at 6.4 V)
CP	0.39, 0.43	83	240	3.1		17	2011^23^	Galvanostatic (7.7 mA/cm^2^)
CP	0.41, 0.45	82	220	3.8		52	2013^21^	Galvanostatic (5.8 mA/cm^2^)
CP	0.31, 0.34	71	231		0.55	≈0.25^b^	2013^22^	Potentiostatic (3.5 V)
CP	0.33, 0.31	74	710	0.14			2015^24^	Voltage scan (peak luminance at 6 V)
Metal-free emitters that harvest both singlets and triplets for emission								
TADF molecules	0.36, 0.38	88	350	4.5	2.1	40	This work	Galvanostatic (7.7 mA/cm^2^)

^a^Proprietary polymer with attached phosphorescent dendrimer but non-disclosed chemical structure.

^b^Estimated from figure in publication.

^c^Note that for devices driven in potentiostatic mode (i.e., by constant voltage) or by a voltage scan, the EQE and current efficacy are most often not recorded at peak luminance.

In this context, organic compounds that emit by the process of thermally activated delayed fluorescence (TADF) are interesting and relevant, since they can harvest both singlet and triplet excitons for light emission while being metal-free^42^. In brief, the electrically generated singlets in a TADF compound can emit “directly” by a process termed “prompt fluorescence”, whereas the electrically generated triplets can be thermally activated (or converted) to the emissive singlet state where emission can take place by a process coined “delayed fluorescence”. A small singlet-triplet energy gap (Δ*E*_ST_) facilitates this thermal activation from the triplet to the emissive singlet state, and this is commonly effectuated by designing the compound with donor and acceptor groups with weak electronic connection. The first metal-free TADF-LEC, which emitted monochrome green emission, was reported by Zysman-Colman and Bolink and co-workers in 2015^43^, and it has been followed by a number of other monochrome TADF-LECs^43–50^. However, we note that no reports on white emission from a metal-free white TADF-LECs are to be found in the scientific literature, although conventional long-range donor-acceptor TADF emitters, on general terms, should represent a good fit for this task because of their characteristically broadband emission spectra ^51–53^.

Herein, we address this issue through our pioneering demonstration of white emission from an LEC device based on solely metal-free TADF emitters. The device design constituted identification and concentration tuning of two color-complementary blue- and orange-emitting TADF guest compounds, two metal-free host compounds that enabled for efficient and appropriately distributed energy transfer to the TADF emitters, and a metal-free ionic liquid electrolyte that functioned as stabile counter-ions during the EDL and electrochemical-doping processes. The selection and tuning of the active-material constituents further aimed for a positioning of the in-situ formed emissive p-n junction in the center of the active material in order to suppress electrode- and dopant-induced emission-quenching processes. The developed TADF-LEC, comprising a single-layer active material and air-stabile electrodes, emits broad-band white emission with a high CRI of 88 and color coordinates of (0.36, 0.38). It is notable that this white emission could be delivered with a bright luminescence of 350 cd/m^2^ at an EQE of 2.11%, and that the broadband emission spectrum was demonstrated to be essentially unaffected by viewing angle, current density, and operational time.

## Results

### Material selection and characterization

**Fig. 1:**
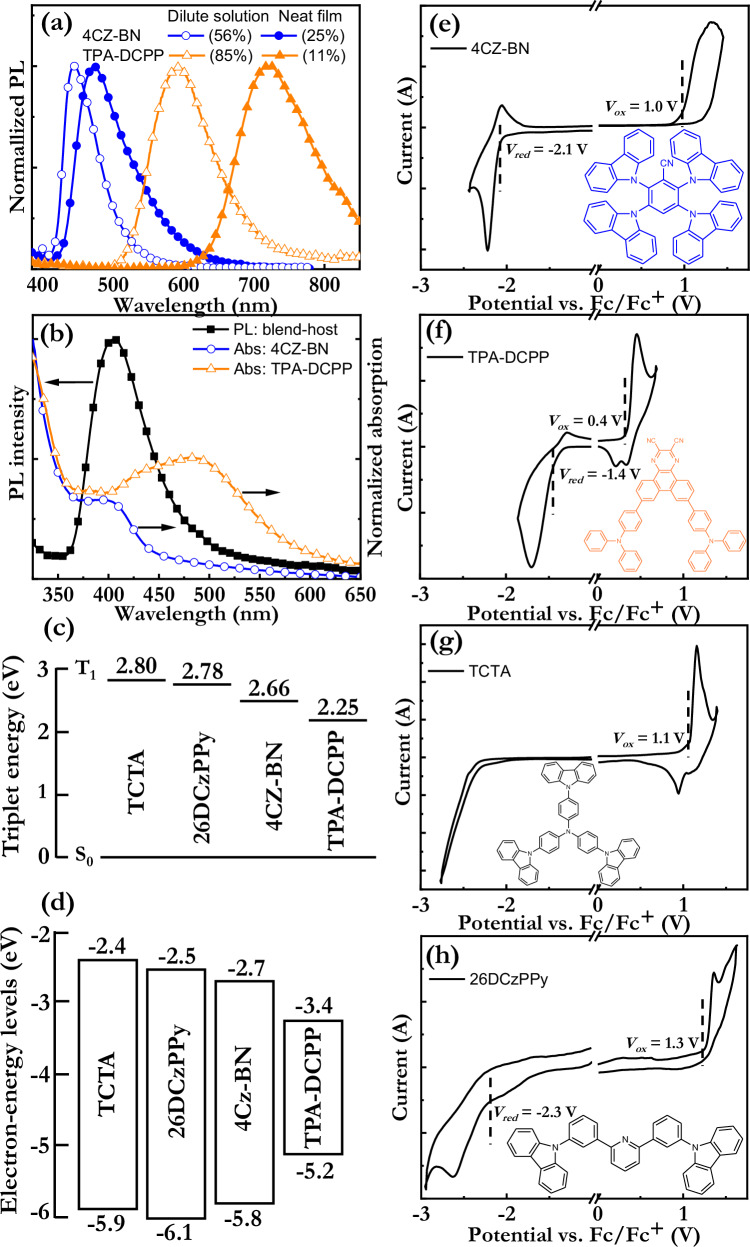
Optical, electronic, and electrochemical properties of the host and guest compounds. **a** The normalized PL spectra of the two TADF emitters in dilute chlorobenzene solution (open symbols, solute concentration = 1 × 10^−3 ^g/l) and as neat film (solid symbols, film thickness = 120 nm). The corresponding PLQY values are included in the parentheses. The excitation wavelength was 300 nm. **b** The PL spectrum of the blend-host neat film and the normalized absorption spectra of the two TADF emitters in dilute chlorobenzene solution. The PL excitation wavelength was 300 nm. **c** The triplet energies of the two host compounds and the two TADF emitters. **d** The electron-energy diagram of the two host compounds and the two TADF emitters, as derived from the neat-film CV data. **e**–**h** CV traces of neat films of (**e**, **f**) the two TADF emitters, and (**g**, **h**) the two host compounds. The vertical dashed lines indicate the derived onset potentials for oxidation and reduction. The corresponding chemical structures are presented in the insets.

The identification of color-complementary TADF emitters with appropriate optoelectronic and electrochemical properties is a crucial exercise in the quest for a white TADF-LEC, and our selected candidates were blue-emitting 2,3,5,6-tetrakis(carbazol-9-yl)benzonitrile (4CZ-BN)^54^ and orange-emitting 7,10-bis(4-(diphenylamino)phenyl)-2,3-dicyanopyrazinophenanthrene (TPA-DCPP)^55^; see insets in Fig. 1e, f for their chemical structures. This selection was motivated by that they feature color-complementary and broadband PL spectra, a small energy gap (∆*E*_ST_) between the first excited singlet level (S_1_) and the first excited triplet level (T_1_) in neat film of ∆*E*_ST_ = 0.22 eV for 4CZ-BN^45^ and ∆*E*_ST_ = 0.13 eV for TPA-DCPP^55^, and since they have been reported of being capable of delivering efficient TADF emission in both dilute solution and as neat film^54,55^.

Figure 1a presents the PL spectra of 4CZ-BN (blue symbols) and TPA-DCPP (orange symbols) in dilute chlorobenzene solution (open symbols) and as neat film (solid symbols). The corresponding PLQY values are displayed in the upper inset. The dilute 4CZ-BN solution exhibits a PL peak of 445 nm, full width at half maximum (FWHM) of the PL envelope of 60 nm, and a PLQY of 56%; whereas the TPA-DCPP solution features a PL peak of 590 nm, a notably broad FWHM of 100 nm, and a high PLQY of 85%. However, we find that the PLQY drops drastically and that the PL envelope redshifts and broadens markedly, when the emitters are transferred from dilute solution to neat film, which implies that they are prone to aggregation-caused quenching (ACQ). The issue with ACQ is particularly problematic for TPA-DCPP, which features a PLQY drop from 85% in dilute solution to 11% in neat film.

A practical method for suppressing the ACQ of an emitter in the neat solid state is by dispersing it into a compatible solid-state host matrix. Functional host-matrix compounds should be soluble in the same type of solvents as the guest emitter(s) to allow for efficient guest dispersibility and solution processability of the host:guest blend. Here, we selected tris(4-carbazoyl-9-ylphenyl)amine (TCTA) and 2,6-bis(3-(carbazol-9-yl)phenyl)pyridine (26DCzPPy) as the host compounds, and their chemical structures are displayed in the insets of Fig. 1g, h. We find that these two hosts, as desired, are highly soluble in the same solvents that dissolve the two emitters, as exemplified by that all four compounds feature a high solubility of >20 g/l in chlorobenzene. It is further important that T_1_ of both TCTA and 26DCzPPy is larger than that of the two TADF emitters, as displayed in Fig. 1c, since this assures that the triplet excitons can be transferred to, and be trapped on, the guest emitters^56,57^.

The functional operation of a LEC relies on ion migration in the active material for the formation of EDLs at the electrode interfaces and electrochemical doping of the host and guest. We selected the ionic liquid tetrahexylammonium tetrafluoroborate (THABF_4_) as the source of the mobile ions since it has been reported to be highly stabile during LEC operation^58,59^. Cyclic voltammetry (CV) was utilized to probe the electrochemical doping capacity of the four prospective host and guest compounds, with THABF_4_ as the source of the dopant counter-ions. Figure 1e–h presents CV traces of neat films of the two TADF-emitters and the two host compounds, while the CV traces of the same compounds in dilute solution are displayed in Supplementary Fig. 1. A qualitatively analysis yields that the two TADF guest emitters and the 26DCzPPy host exhibit relatively reversible oxidation and reduction reactions, while the TCTA host exhibits partially reversible oxidation but irreversible reduction.

The onset potentials for the electrochemical oxidation and reduction reactions (*V*_Ox/Red_) are indicated by, and displayed next to, the vertical dashed lines in Fig. 1e–h and S1. By assuming that the observed reversible oxidation and reduction reactions correspond to electrochemical p-type and n-type doping, respectively, of the emitter/host, it is possible to translate the measured onset potentials to the HOMO and LUMO energy levels of the guest emitters and the host with the aid of the following equation: *E*_HOMO/LUMO_ (eV) = −*e*(4.8 V + *V*_Ox/Red_), where *e* is the elementary charge^60^. The LUMO level of TCTA was derived by adding the optical bandgap energy (i.e., its onset energy for absorption of 3.5 eV) to its HOMO level.

Figure 1d presents the derived electron-energy diagram of the two host compounds (left) and the two TADF guest emitters (right), as gleaned from the neat-film CV data. The corresponding electron-energy levels derived from the solution-CV data were found to differ by a mere 0–0.2 eV (compare neat-film-derived redox potentials in Fig. 1e–h with solution-derived redox potentials in Supplementary Fig. 1), and this small deviation between neat film and dilute solution implies that the HOMO and LUMO levels of these host and guest-emitter compounds are relatively unaffected by molecular packing and aggregation effects. We find that the HOMO and LUMO levels of the two host compounds, as desired, encompass the HOMO and LUMO levels of both TADF emitters, which implies that both the hole and the electron will be trapped on the TADF emitters.

Supplementary Fig. 2a displays the PL spectra of neat films of TCTA, 26DCzPPy, and the TCTA:26DCzPPy (1:1 by mass) blend-host. The marked redshift of the PL spectrum of the blend-host film compared to the PL spectra of the two single-host films suggests that the emissive species of the blend-host film is an exciplex. This is in line with the electron-energy diagram in Fig. 1d shows that the hole is preferentially localized on the TCTA donor, while the electron is localized on the 26DCzPPy acceptor. Supplementary Fig. 2b presents the PL transient of the blend-host film, and the observation of a distinct ns-short prompt-fluorescence component (emissive lifetime = 2.4 ns) and a distinct µs-slow delayed-fluorescence component (emissive lifetime = 0.74 µs) provides support for that the blend-host film forms an exciplex that emits by the TADF process.

Figure 1b reveals that the PL spectrum of the TCTA:26DCzPPy blend-host film (solid black squares) overlaps the absorption spectra of the two TADF emitters in dilute chlorobenzene solution. The significant overlap between the emission of the blend-host matrix and the absorption of “isolated” TADF emitters establishes that a basic requirement for a Förster resonance energy transfer (FRET) from the blend-host to the guest emitters is fulfilled.

In order to suppress efficiency-limiting exciton-polaron and exciton-electrode quenching, the emissive p-n junction should be positioned close to the center of the active material in LEC devices. It has been demonstrated that this can be effectuated in host:guest LECs through a balancing of the electron and hole “traps” and the electron and hole mobility on the host matrix^45,61^. The trap depth for electron (hole) transport is herein defined to be equal to the offset between the LUMO (HOMO) values of the host and the guest, with the effective LUMO (HOMO) of a blend-host being equal to the lowest LUMO (highest HOMO) of its two constituents. With the data in Fig. 1d at hand, we derive that the electron and hole trap depths for the 4CZ-BN guest in the blend-host matrix are 0.2 and 0.1 eV, respectively; whereas the electron and hole trap depths for the TPA-DCPP guest emitter in the blend-host matrix are 0.9 and 0.7 eV, respectively. We also call attention to that the employed TCTA:26DCzPPy blend-host material (mass ratio = 1:1) has been reported to exhibit a relatively balanced electron/hole mobility^45^. Thus, we anticipate that the dynamically formed p-n junction will be centered in the active material and that exciton quenching losses due to the proximity of metal electrodes and dopants, as a consequence, should be suppressed.

### Design and tuning of the active material for white emission

**Fig. 2:**
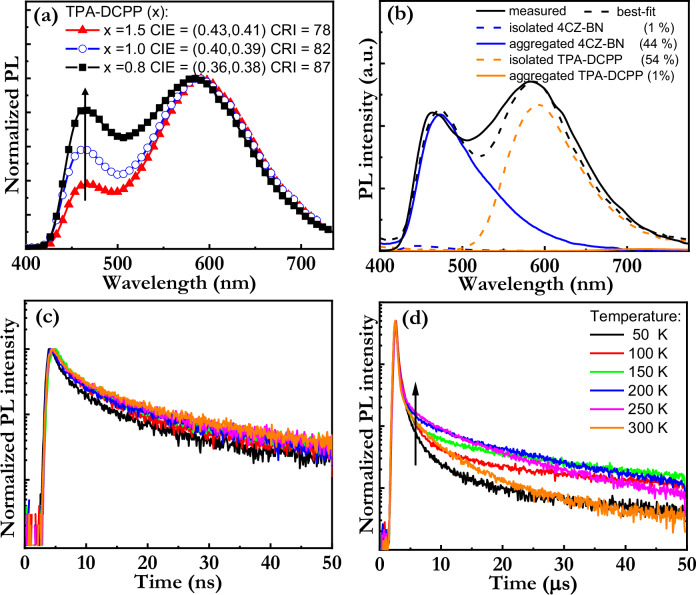
Tuning and analysis of the active material for white emission. **a** The normalized PL spectra of thin films of the TCTA:26DCzPPy:4CZ-BN:TPA-DCPP:THABF_4_ active material, with the mass ratio being 50:50:50:x:10. The arrow indicates decreasing concentration of TPA-DCPP, as detailed in the inset. **b** The measured (solid black line) and the best-fitted (dashed black line) PL spectrum of the optimized white-emitting active material (*x* = 0.8). The fit was performed by varying the relative weight of four PL spectral components (derived from Fig. 1a): “isolated 4CZ-BN”, “aggregated 4CZ-BN”, “isolated TPA-DCPP”, and “aggregated TPA-DCPP”; the best-fit weights of these spectral components are included in the inset. The PL transients of a thin film of the optimized white-emitting active material at different temperatures (see inset in **d**) in the time regime of (**c**) prompt fluorescence and (**d**) delayed fluorescence. The PL is detected in the wavelength range of 400–700 nm. The arrow in (**d**) indicates increasing temperature.

We now shift our focus to the tuning of the relative concentrations of the five constituents in the TCTA:26DCzPPy:4CZ-BN:TPA-DCPP:THABF_4_ active material for high-performance broadband-white LEC operation. First, the mass ratio of the TCTA:26DCzPPy blend-host constituents was set to 50:50 in order to obtain balanced electron and hole mobility^45^. Thereafter the preferred mass ratio of the THABF_4_ ionic liquid was determined to be TCTA:26DCzPPy:THABF_4_ 50:50:10 in order to achieve facile device turn-on and stabile LEC operation^45^. The optimum relative concentration for the blue-emitting 4CZ-BN guest emitter was by systematic device investigation (see Table S1) established to be the same as each of the two blend-host constituents, i.e., the TCTA:26DCzPPy:4CZ-BN:THABF_4_ mass ratio was determined to be 50:50:50:10^45^. Figure 2a presents the final PL investigation that resulted in that a mass ratio of the orange-emitting DPA-DCPP of 0.8 enabled for the highest quality broad-band white emission with a high color rendering index (CRI) of 87 and CIE coordinates of (0.36, 0.38). Combined, these procedures thus resulted in the optimum mass ratio for the active material was TCTA:26DCzPPy:4CZ-BN:TPA-DCPP:THABF_4_ = 50:50:50:0.8:10.

A comparison between the PL spectra of thin films of the blend-host (Fig. 1b, solid black squares) and the optimized active material (Fig. 2a, solid black squares) reveals that the blend-host does not contribute with any detectable PL in the optimized active material, which in turn establishes that the energy transfer from the blend-host to the two TADF emitters is complete in the optimized active material. This consequently suggests that the constituents are well mixed, which is in agreement with that the optimized active material exhibits a highly flat surface, without any indications of phase separation, in atomic force microscopy (see Supplementary Fig. 3).

Nevertheless, the observed distinct difference between the PL spectrum of the emitters in “isolated” form (i.e., in dilute solution) and in “aggregated” form (i.e., in neat film) in Fig. 1a enables for an analysis of the physical state (“isolated” or “aggregated”) of the two emitters in the optimized dry active material. Figure 2b presents the measured (solid black line) and the best-fit simulated (dashed black line) PL spectrum of the active material, with the four fitting PL spectral components being “isolated 4CZ-BN”, “aggregated 4CZ-BN”, “isolated TPA-DCPP”, and “aggregated TPA-DCPP”, as imported from Fig. 1a. This fitting shows that the white PL spectrum essentially only comprises PL from aggregated 4CZ-BN and isolated TPA-DCPP, which is in good agreement with that 4CZ-BN exists in a significant mass fraction of 31% in the optimized active material while the mass fraction of TPA-DCPP is very low at 0.5%. Figure 2c, d presents the temperature dependence of the PL transient of the optimized active material in the time regimes of prompt fluorescence and delayed fluorescence, respectively. The latter PL transient is observed to be temperature-activated at delay times shorter than 5 µs (see arrow in Fig. 2d), which is in line with the active material emits by the process of TADF. More specifically, the RISC rates from the (non-emissive) T_1_ states to the (emissive) S_1_ states are temperature activated, and the time required for these activation steps is manifested in that the fluorescence is delayed. These convoluted data are however difficult to analyze further since the measured emission originates from two guest emitters, and since a host-to-guest energy transfer often precedes the actual emission process.

**Fig. 3:**
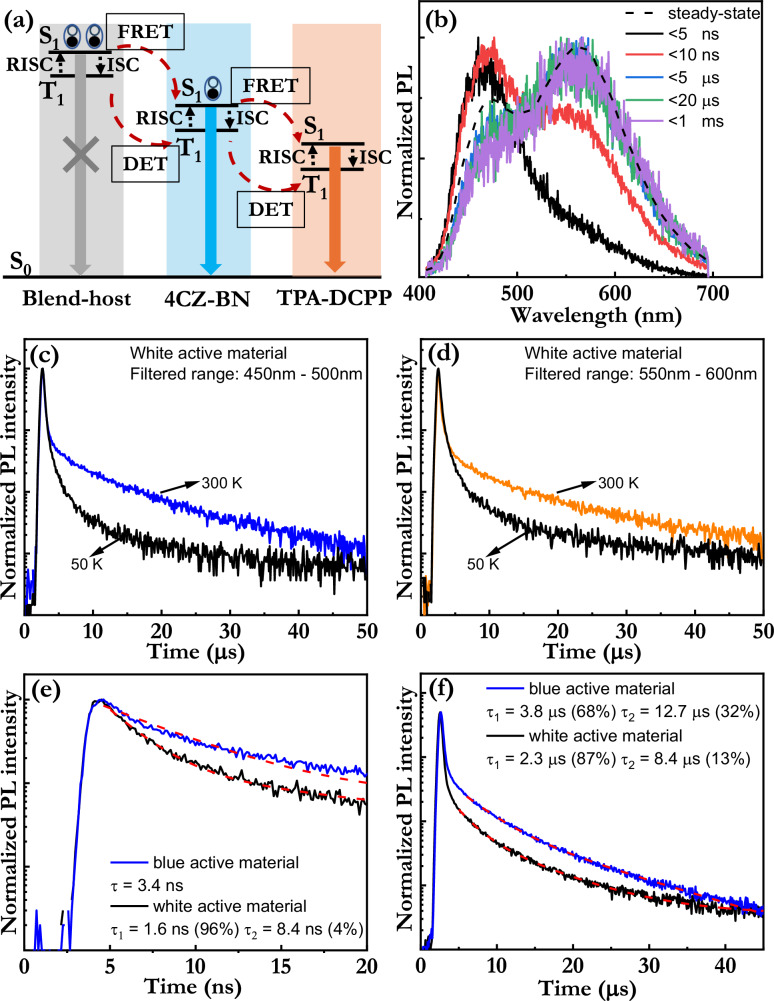
Energy transfer and emission of the active material. **a** Schematic presentation of key energy-transfer and emissive processes in effect in the optimized white active material. **b** The PL spectrum of the white active material as a function of the detection time after photoexcitation, with the detection time identified in the inset. The steady-state PL spectrum (dashed black line) is included for comparison. The thickness of the active-material film was 120 nm, and the laser excitation wavelength was 337 nm. PL transients of the white active material at 50 K and 300 K, for the filtered wavelength range of (**c**) 450–500 nm and (**d**) 550–600 nm. The measured (solid lines) and the fitted (dashed red lines) PL transients of the white-emitting active material and a blue-emitting active material in the time regime of (**e**) prompt fluorescence and (**f**) delayed fluorescence, with the active material identified in the insets. The fits were performed with either a single-exponential or a bi-exponential function, and the derived emissive lifetime and the weight of the fitting components are presented in the insets. The PL transients in (**e**, **f**) were recorded at 300 K.

Thus, to facilitate a more detailed analysis and discussion, we present a schematic in Fig. 3a that identifies the key energy-transfer and emission processes in effect in the optimized white active material. Photoexcited excitons are primarily formed on the blend-host (Fig. 3a, left part) and the blue emitter (middle part), due to their relative high concentration in the active material (and their efficient absorption at the employed excitation wavelengths; see Fig. 1b and Supplementary Fig. 4). The previously observed absence of blend-host emission in the white active material (cf. Fig. 2a with Fig. 1b) reveals that the blend-host excitons are efficiently transferred, before light emission, to the two emitters by either FRET, or Dexter energy transfer (DET), or a combination thereof (see also Fig. 1b, c and associated discussion).

Figure 3b presents the PL spectrum of the white active material as a function of the detection time after the end of photoexcitation, with the steady-state PL spectrum (dashed black line) included as a reference. It is interesting that it is only the blue emitter that contributes with photons at short detection times (<5 ns) after photoexcitation. This implies that the blue emitter is photoexcited and emitting; but also that the blend-host singlet excitons are almost solely transferred to the blue (and not the orange) emitter, which is in line with it being 60 times more prevalent than the orange emitter in the optimized white active material. At an intermediate detection time, ranging between 5 and 10 ns, the orange emission begins to become significant, and at longer detection times, in the microsecond time regime, it is dominating. This suggests that a cascaded exciton transfer, in the form of blend-host→blue-emitter→orange-emitter or blue-emitter→orange-emitter, has preceded the formation of the orange excitons. We note that the steady-state PL spectrum, as expected, represents a combination of the short-time PL spectrum and the long-time PL spectrum, but that the long-time PL spectrum has the larger relative influence.

In order to investigate whether both of the emitters in the white active material emit by the TADF process, we have measured PL transients with a selective-wavelength filter positioned between the sample and the detector. Figure 3c presents the PL transients recorded at 50 K and 300 K when the filter only allows blue photons with wavelengths between 450 and 500 nm to reach the detector; whereas Fig. 3d is measured with another filter that only allows orange photons with wavelengths between 550 and 600 nm to pass. The observation of thermally activated delayed fluorescence in both the blue and the orange wavelength range shows that both emitters effectively emit by the TADF mechanism, i.e., that triplets have been thermally excited to the emissive singlet state before both the blue and the orange emission.

The final energy-transfer step between the blue and the orange emitter was studied by comparing the PL transient of the white active material with the PL transient of a blue-emitting active material, which is distinguished from the white material through the removal of the orange emitter. Figure 3e, f presents the measured (solid line) and the best-fitted (red dashed line) PL transients in the time regime of prompt fluorescence and delayed fluorescence, respectively. The fits were performed with either a single-exponential or a bi-exponential function, and the derived emissive lifetimes and the weight of the fitting components are presented in the insets.

Figure 3e shows that the blue-emitting material exhibits a single prompt fluorescence lifetime of 3.4 ns, but that the addition of the orange emitter results in a notable shortening of this lifetime (of the majority component) to 1.6 ns and to the emergence of a minor slower component. We attribute the shortening of the major prompt (blue) emissive lifetime, following the addition of the orange emitter, to that a significant fraction of the blue singlet excitons are transferred to the orange emitter by FRET. The emergence of the minor, slower component in the white active material is then due to the prompt emission of this FRET-transferred orange singlets. This conclusion is supported by that orange light emission begins to be observed at times between 5 and 10 ns in Fig. 3b.

Figure 3f shows that the blue active material exhibits two delayed fluorescence components, which should be effectively determined by the time required for the execution of an ISC/RISC cycle on either the blend host or on the blue emitter. We tentatively assign the faster-delayed fluorescence component to the execution of one ISC/RISC cycle on the blend host, due to the observation that the neat blend-host material exhibits a short delayed fluorescence lifetime of <1 µs (see Supplementary Fig. 2b and related discussion). The addition of the orange emitter will result in that some of the blue delayed fluorescence is lost to energy transfer to the orange emitter, and we find that the opening of this additional energy-transfer channel results in a slight shortening of the two emissive lifetimes and to a relative redistribution to the faster of the delayed fluorescence components. We mention that we deem it unlikely that we detect the execution of an ISC/RISC cycle directly on the orange emitter since it has been reported to feature an order-of-magnitude longer delayed emissive lifetime in similar systems^55^, and since this should be manifested in the (non-observed) emergence of a third delayed fluorescence component. Importantly, the overall take-home message of our optical analysis of the active material is that the energy transfer to the two TADF emitters should be efficient and relatively balanced.

### LEC device development

**Fig. 4:**
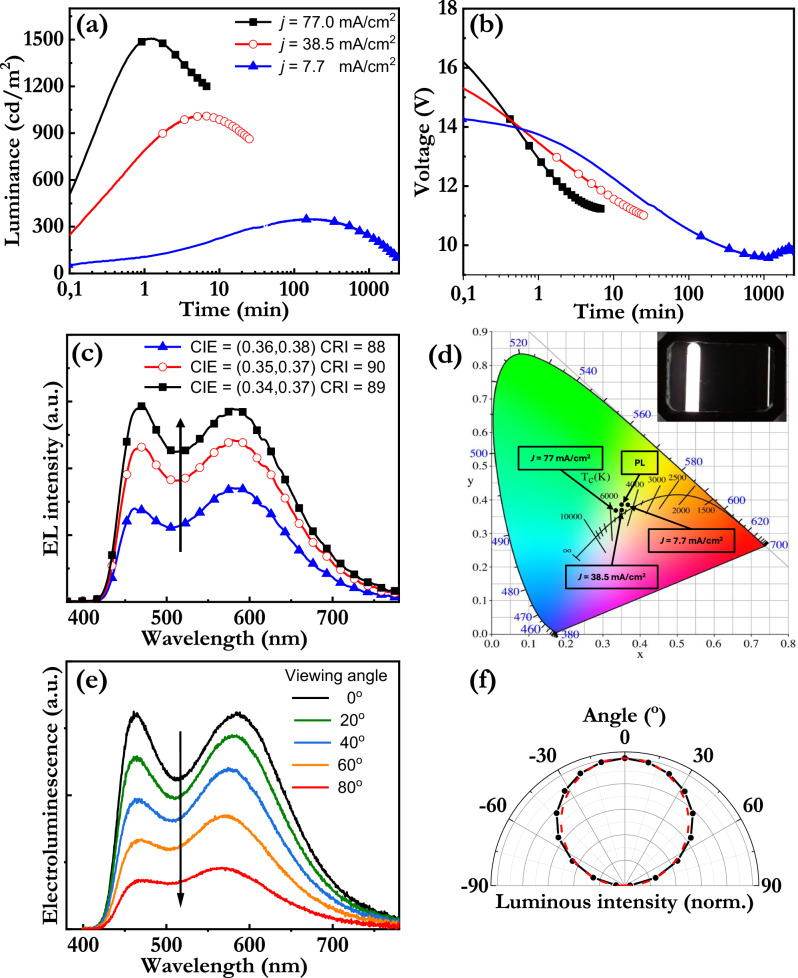
Device performance of the white TADF-LEC. The temporal evolution of (**a**) the luminance, and (**b**) the voltage of representative pristine white TADF-LECs during electrical driving by three different current densities, as identified in the inset in (**a**). **c** The EL spectrum (at peak luminance) for the different current densities, with the arrow indicating increasing current density. The corresponding color metrics are presented in the inset. **d** The 1931 CIE color space chromaticity diagram, with the position of the white TADF-LEC at three different current densities as well as the PL spectrum of the optimized active material included. The inset presents a photograph of the uniform white emission from the TADF-LEC during driving by *j* = 7.7 mA/cm^2^. **e** The steady-state EL spectrum of the white TADF-LEC as a function of viewing angle, as identified in the inset. The arrow indicates increasing viewing angle. **f** The steady-state luminous intensity of the white TADF-LEC as a function of viewing angle (solid black circles and solid black line), with the luminous intensity of an ideal Lambertian emitter indicated by the red dashed line.

We therefore now turn to the investigation of the merit of the PL-optimized white active material in LEC devices, which are equipped with air-stabile electrodes in the form of an indium-tin-oxide/poly(3,4-ethylenedioxythiophene):poly(styrene sulfonate) anode and an Al cathode. Figure 4a,b display the temporal evolution of the luminance and the voltage, respectively, of such representative pristine TADF-LECs (with an active-material thickness of 120 nm) during driving by three different current densities, as detailed in the inset of Fig. 4a. Figure 4c shows the corresponding EL spectra of the TADF-LECs (recorded at peak luminance), with the CIE color coordinates and the CRI values included in the inset; while Fig. 4d presents the CIE data within the 1931 CIE color space chromaticity diagram, with the correlated color temperature (CCT) included as well (see Table 2 for precise values). The PL color coordinates of the white active material are also included in the diagram for reference.

**Table 2:** Summary of the device performance of the tuned white TADF-LEC as a function of the current density

Current density (mA/cm^2^)	EL wave-length peaks (nm)	White-light performance	Turn-on time (to > 100 cd/m^2^) (s)	Peak luminance (cd/m^2^)	Minimum voltage (V)	Current efficacy (cd/A)	EQE (%)
7.7	460, 580	CIE: 0.36, 0.38 CRI: 88 CCT: 4415 K	45	350	9.6	4.55	2.11
38.5	460, 580	CIE: 0.35, 0.37 CRI: 90 CCT: 4745 K	3	1010	10.9	2.63	1.22
77	460, 580	CIE: 0.34, 0.37 CRI: 89 CCT: 4915 K	3	1505	11.2	1.96	0.91

We first call attention to that the TADF-LEC delivers high-quality white light emission, as demonstrated by the high CRI of 88-90, the broadband EL spectrum, and the positioning of the CIE coordinates within the white region of the CIE color space chromaticity diagram. The inset in Fig. 4d presents a photograph of the uniform and “natural daylight” appearing white emission that is delivered by the TADF-LEC, which is in line with the derived CCT values of 4400–5000 K, as detailed in Table 2. Figure 4e, f further show that both the EL spectrum and the perceived luminance of the white TADF-LEC device (with an active-material thickness of 120 nm) are highly invariant to the viewing angle, with the latter demonstrating that this white TADF-LEC functions as a Lambertian emitter. These observations thus serve as evidence for that the optical design of the LEC device for quality white emission has been successful.

From a device-operation viewpoint, we note with interest that the white TADF-LEC exhibits increasing luminance and decreasing voltage during the initial turn-on period for all current densities (Fig. 4a, b), and we call attention to that these are characteristic indicators of the formation of EDLs and a p-n junction doping structure by electrochemical doping in LEC devices^62,63^. These observations therefore provide support for that the ionic-liquid electrolyte contributes with mobile ions in the active material, and that these mobile ions can function as the dopant counter-ions during the electrochemical p- and n-type doping of the blend-host and the TADF emitters. The electrochemically formed p- and n-doping regions grow from the anode and cathode, respectively, and meet to form a p-n junction in the bulk of the LEC active material. We determined the position of the “emissive p-n junction” in the active material, or more specifically the average position of exciton generation, by a combined measurement and simulation of the EL spectrum as a function of the viewing angle, using a procedure described in the experimental section and with the fitting results depicted in Supplementary Fig. 5^61^. We find that the emissive p-n junction is well separated from both electrodes (albeit positioned slightly closer to the reflective cathode) at steady state in the white TADF-LEC, which is important since it implies that the non-desired electrode- and dopant-induced quenching of the excitons is suppressed. This in turn yields that our design of the active material with balanced electron and hole traps and balanced electron and hole mobility on the host has fulfilled its purpose.

The functional device design is finally manifested in that the white TADF-LEC delivers a respectable light-emission performance, as summarized in Table 2. The turn-on time to a luminance exceeding 100 cd/m^2^ is relatively fast at a few seconds, and the EQE is 2.11% (corresponding to a current efficacy of 4.55 cd/A) at a bright luminance of 350 cd/m^2^ during galvanostatic driving by a current density of 7.7 mA/cm^2^. The peak luminance can be increased to exceed 1500 cd/m^2^ by simply increasing the driving current density to 77 mA/cm^2^. The measured operational lifetime of 40 h above 100 cd/m^2^, albeit far from the record for LEC devices^64^, is still long for a device comprising TADF compounds for the emitting species. It is further notable and attractive that the broadband white EL spectrum remains essentially invariant during this long-term operation (see Supplementary Fig. 6), which suggests that phase separation or selective degradation of one of the two TADF emitters are not primary degradation factors.

## Discussion

We conclude by briefly comparing the performance of the herein-developed white TADF-LEC with the state-of-the-art for white-emitting LEC devices, as summarized in Table 1. Our measured CRI of 88–90 is higher than earlier reports, with the notable exception being the study by Nishide and co-workers which reported an impressive CRI of 96 with the LEC emitter being a blend of an Ir complex and a conjugated polymer^41^. However, a drawback with their device was that the EQE and operational lifetime were very modest^41^. The highest values for the EQE have, as expected, been reported with phosphorescent Ir complexes as the emitter, and the current record of 4.7% belongs to Qiu and co-workers^27^. For LECs based on more sustainable metal-free emitters, the record EQE of 2.4% does, somewhat surprisingly, still originate from the original report of white-emission from LECs by Yang and Pei^18^, although our obtained EQE of 2.11% is close behind. In this context, we mention that the first white LEC (in agreement with several of the other studies presented in Table 1) was driven in potentiostatic (i.e., constant voltage) mode and that it is common that the peak EQE is preceding the peak luminance in time in LECs during constant-voltage (or scan-voltage) driving because of their dynamic electrochemical doping process^65^. During galvanostatic (i.e., constant-current) operation, as utilized in our study, the peak luminance and peak EQE must coincide in time. We also note that the herein-reported operational lifetime (above 100 cd/m^2^) of 40 h is similar to the state-of-the-art for white LECs at 52 h. We mention that a lowering of the drive voltage most likely will result in a further improvement of the operational stability (and the power conversion efficiency), and we speculate that this could be achieved by an increase in the electron and hole mobility of the blend guest, a lowering of the electron and hole traps, or a combination thereof.

In summary, we report on the first demonstration of white emission from a LEC device, which solely employs metal-free TADF compounds as the emissive species. This development constituted a systematic design, investigation, and tuning of the energy-transfer processes and the electrochemically formed doping structure within the metal-free LEC active material, which comprises two color-complementary blue and orange TADF emitters, a blend host, and an ionic liquid electrolyte. The white TADF-LEC delivers fast-to-turn-on white emission with a high color rendering index of 88 at an external quantum efficiency of 2.11% and a luminance of 350 cd/m^2^, and the broadband emission spectrum is demonstrated to be invariant to viewing angle, current density, and operational time. A comparison with the literature reveals that the attained white LEC performance is competitive with the state-of-the-art. In consideration of that a major merit of the LEC technology is that flexible devices can be fabricated by cost- and energy-efficient ambient-air printing, our study is important in that it demonstrates that practical white LECs can be fabricated with efficient and sustainable emitter materials.

## Methods

### Materials and inks

The TADF emitters 2,3,5,6-tetrakis(carbazol-9-yl)benzonitrile (4CZ-BN, Lumtec) and 7,10-bis(4-(diphenylamino)phenyl)- 2,3-dicyanopyrazino-phenanthrene (TPA-DCPP, Lumtec), the host compounds tris(4-carbazoyl-9-ylphenyl)amine (TCTA, Lumtec) and 2,6-bis(3-(9Hcarbazol-9-yl)pyridine) (26DCzPPy, Lumtec), and the ionic-liquid electrolyte tetrahexylammonium tetrafluoroborate (THABF_4_, Merck) were all used as received. The master inks were prepared by separately dissolving the TADF emitters and the host compounds in chlorobenzene (99.8%, Merck) in a concentration of 37.5 g L^−1^, and thereafter stirring at 70 °C for >6 h on a magnetic hot plate. The active-material inks were prepared by blending the master inks in a desired mass ratio, and thereafter stirring at 70 °C for >2 h on the magnetic hot plate.

### Material characterization

The thin films for the absorption and the photoluminescence (PL) measurements were spin-coated from the master and active-material inks onto carefully cleaned quartz substrates (thickness = 1.0 mm, Alfa Aesar). The absorption and PL spectra were measured with a spectrometer connected to an integrating sphere (C9920-02G, Hamamatsu Photonics). The PL transients were recorded on spin-coated thin films on Si substrates under vacuum, using a Nd-YAG laser (MPL15100-DP-TH, QS LASERS), which delivers 600 ps excitation pulses with a wavelength of 355 nm at a repetition rate of 20 Hz, as the excitation source, and a streak camera (C4334, Hamamatsu Photonics) for the detection. The cyclic voltammetry (CV) measurements were carried out with a computer-controlled potentiostat (Autolab PGSTAT302, driven by the GPES software). For the thin-film CV, the working electrode comprised the material-under-study drop-cast on a Au-covered glass substrate; while for the solution CV, a Pd disc (diameter = 5 mm) was the working electrode, and the material-under-study was dissolved in the electrolyte solution at a concentration of 1–5 g/l. For both CV measurements, a Pt rod was the counter electrode, an Ag wire was the quasi-reference electrode, and 0.1 M THABF_4_ (Merck) in anhydrous CH_3_CN/DMF was the electrolyte solution. Directly after each CV scan, a calibration scan was run with a small amount of ferrocene added to the electrolyte solution. All CV potentials are reported vs. the ferrocene/ferrocenium ion (Fc/Fc^+^) reference potential. The reduction/oxidation onset potentials are defined as being equal to the intersection potential between the baseline and the tangent of the current at its half-peak value. The CV sample preparations and measurements were performed in a N_2_-filled glove box ([O_2_] <2 ppm, [H_2_O] <1 ppm).

### Device fabrication and measurements

For the device fabrication, the indium-tin-oxide (ITO) coated glass substrates (glass thickness = 0.7 mm, glass area =  15 × 15 mm^2^, ITO area = 15 × 10 mm^2^, *R*_sheet_ = 20 Ω sq^−1^, Kintec) were cleaned by sequential ultrasonication in alkaline cleaning solution, acetone, and 2-propanol. A poly(3,4-ethylenedioxythiophene):poly(styrene sulfonate) (PEDOT:PSS) ink (Clavius P VP AI 4083, Heraeus) was spin-coated onto the ITO at 4000 rpm for 60 s (acceleration =  1000 rpm/s), and the coated PEDOT:PSS film was dried at 120 °C for 30 min. The active-material ink was spin-coated on the dry PEDOT:PSS layer at 2000 rpm for 60 s (acceleration = 2000 rpm/s), and the coated active-material film was dried at 70 °C for 2 h. The dry thickness of the PEDOT:PSS and the active-material films was 40 nm and 120 nm, respectively, as measured with a profilometer (DekTak XT, Bruker). Finally, 100 nm thick Al cathodes were deposited on top of the active material by thermal vacuum evaporation (at *p* < 5 × 10^−6^ mBar) through a shadow mask, with the deposition rate being 1–6 Å/s. The 0.85 × 0.15 cm^2^ emission area was defined by the overlap between the pre-patterned ITO anode and the shadow-mask patterned Al cathode.

The LEC devices were electrically driven and measured by a computer-controlled source-measure unit (Agilent U2722A). The luminance was measured with a calibrated photodiode, equipped with an eye-response filter (Hamamatsu Photonics), and connected to a data acquisition card (National Instruments USB-6009) via a current-to-voltage amplifier. The EL spectrum was measured with a calibrated spectrometer (USB2000+, Ocean Optics). All of the above-presented fabrication and measurement procedures, with the exception of the deposition of PEDOT:PSS, were carried out in two interconnected N_2_-filled glove box ([O_2_] <2 ppm, [H_2_O] <1 ppm).

For the determination of the position of the emissive p-n junction, the angle-dependent EL spectrum and intensity were measured with a custom-built, automated spectrogoniometer setup. The LEC was driven by a constant current density of 75 mA/cm^2^, and the EL data measured after 3 min of operation. The simulation of the same EL data was performed with a commercial software (Setfos version 5.2, Fluxim AG, Switzerland). The active material was simulated as transparent and with a wavelength-dependent refractive index. The latter was estimated as the effective medium of the four active-material constituents, with the wavelength-dependent refractive index of TCTA, 26DCzPPy, and 4CZBN being determined by ellipsometry (UVISEL Plus, HORIBA) while that of THABF_4_ was set to *n* = 1.42 over the entire spectral range. Supplementary Fig. 7 presents the wavelength-dependent refractive indices for the active material and its four constituents. The distribution of the emissive dipoles was represented by a Gaussian function with a FWHM of 22% of the total active-material thickness, while their orientation was considered isotropic. The intrinsic emission spectrum of the emissive dipoles in the active material was set equal to the measured PL spectrum of the optimized white active-material film. The position of the emissive dipoles, i.e., the center position of the emissive p-n junction, in the active material was determined by minimizing the root mean square error between the simulated and the measured angle-dependent EL data, using a previously published procedure ^61^.

## Supplementary information

Supplementary Information

Transparent Peer Review file

## Data Availability

The raw data generated in this study have been deposited in the figshare database and can be downloaded at: https://figshare.com/s/7a2743d1b4b633baa2f3.
