# Supplementary material for: White light-emitting electrochemical cells based on metal-free TADF emitters
Source: Nat Commun. 2025 Jan 14;16:653. doi: 10.1038/s41467-025-55954-3 (PMC11733125; doi:10.1038/s41467-025-55954-3)
Supplement: Supplementary file 1 — Supplementary Information [file 41467_2025_55954_MOESM1_ESM.pdf]

Supplementary Information:

White Light-Emitting Electrochemical Cells Based on Metal-Free TADF emitters

Shi Tang[1,2] Youichi Tsuchiya,[3] Jia Wang,[1] Chihaya Adachi,[3] and Ludvig Edman[1,2,4]*

[1] The Organic Photonics and Electronics Group, Department of Physics, Umeå University, SE-90187 Umeå, Sweden

[2] LunaLEC AB, Linnaeus väg 24, SE-90187 Umeå, Sweden

[3] Center for Organic Photonics and Electronics Research (OPERA), Kyushu University, 744 Motooka, Nishi-ku, Fukuoka 819-0395, Japan

[4] Wallenberg Initiative Materials Science for Sustainability, Department of Physics, Umeå University, SE-90187 Umeå, Sweden

Correspondence: Ludvig Edman (e-mail: ludvig.edman@umu.se)

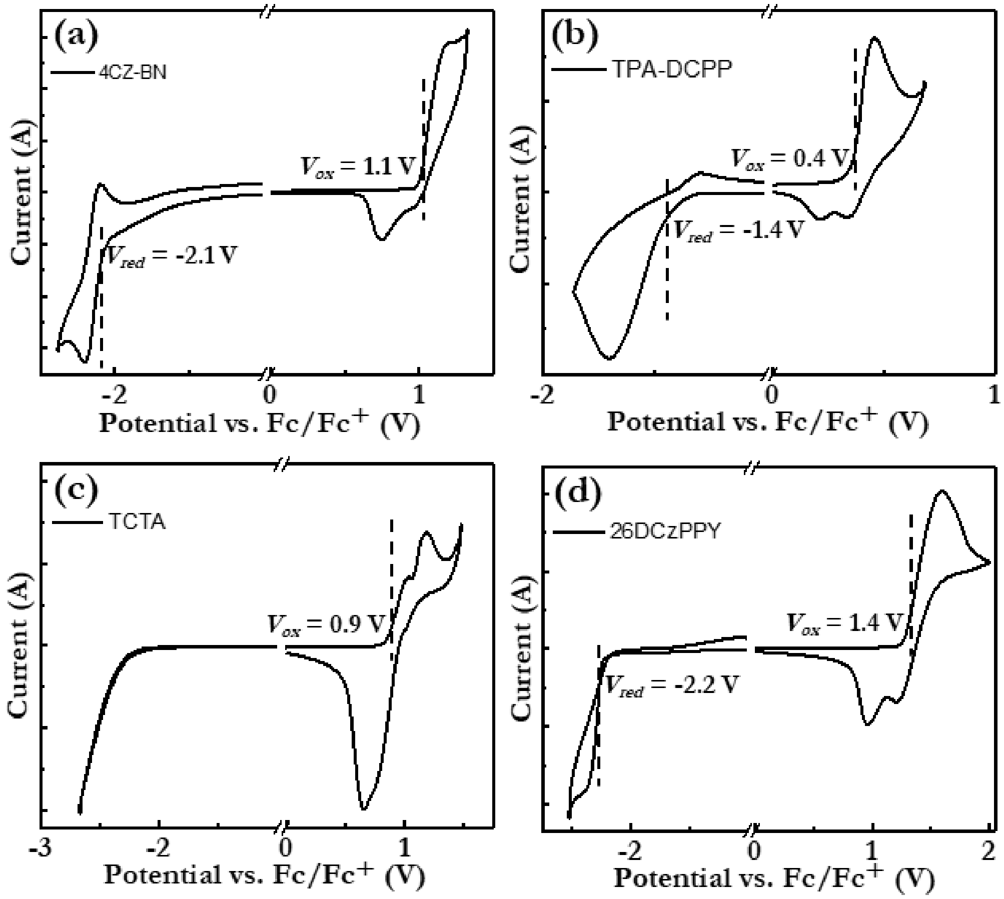

Supplementary Figure 1. Electrochemical properties of the guest and host compounds in solution. Cyclic voltammetry traces of (a,b) the two TADF emitters and (c,d) the two host compounds in DMF solution, with the solute concentration being ~1g/l.

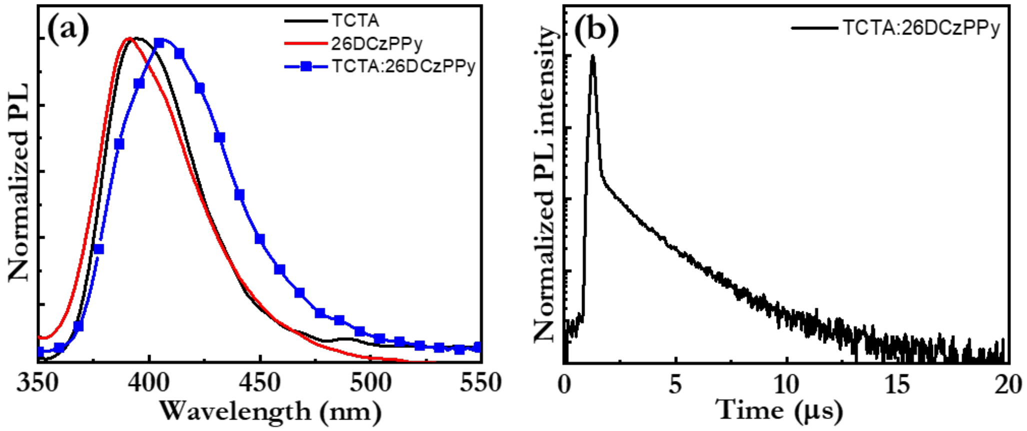

Supplementary Figure 2. Optical analysis of the blend-host matrix. (a) The normalized PL spectra of neat films of the TCTA host (solid black line), the 26DCzPPY host (solid red line) and the TCTA:26DCzPPY (1:1 mass ratio) blend-host (solid blue squares). (b) The room-temperature PL transient of a blend-host (1:1 mass ratio) film, with a thickness of 120 nm. The excitation wavelength was 300 nm for the recording of the PL spectrum and 337 nm for the PL transient.

Supplementary Table 1. The performance of the blue-emitting TCTA:26DCzPPy:4CZ-BN:$THABF_4$ LEC as a function of the relative concentrations of the 4CZ-BN guest emitter and the $THABF_4$ ionic liquid. The mass ratio of the TCTA:26DCzPPy blend-host was locked at 50:50, and the LECs were driven by a constant current density of 7.7 mA/cm^2.

4CZ-BN mass ratio	$THABF_4$ mass ratio	Turn-on time (>100 cd/m^2) (s)	Peak luminance (cd/m^2)	Current efficacy (cd/A)	EQE (%)	Lifetime (>100 cd/m^2) (h)
10	10	---	11	0.14	0.07	---
30	10	---	83	1.1	0.5	---
45	10	5	205	2.7	1.3	3.1
50	10	6	250	3.3	1.6	11.5
70	10	25	189	2.5	0.7	18.7
50	5	70	164	2.1	1.1	1.6

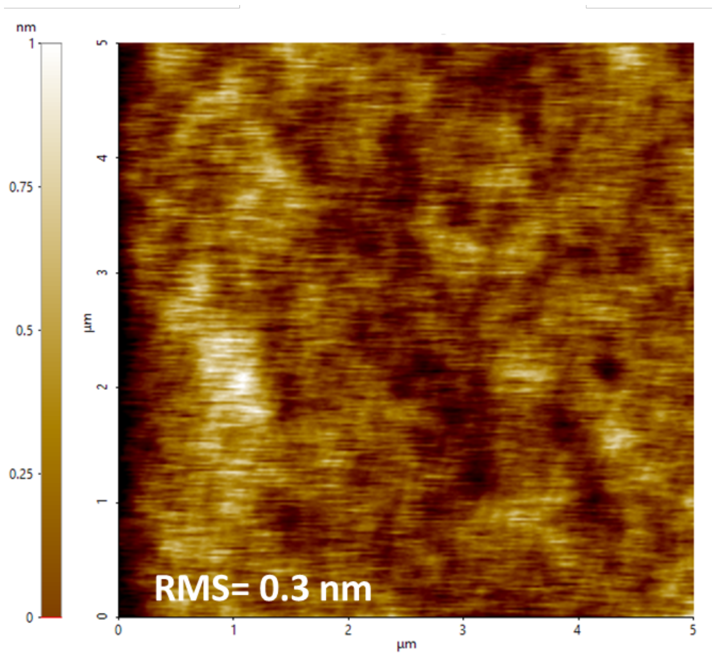

Supplementary Figure 3. Surface morphology of the white active material. 5×5 μm^2 surface topography image of the highly flat (RMS = 0.3 nm) optimized white-emitting active material, as recorded by AFM in non-contact mode.

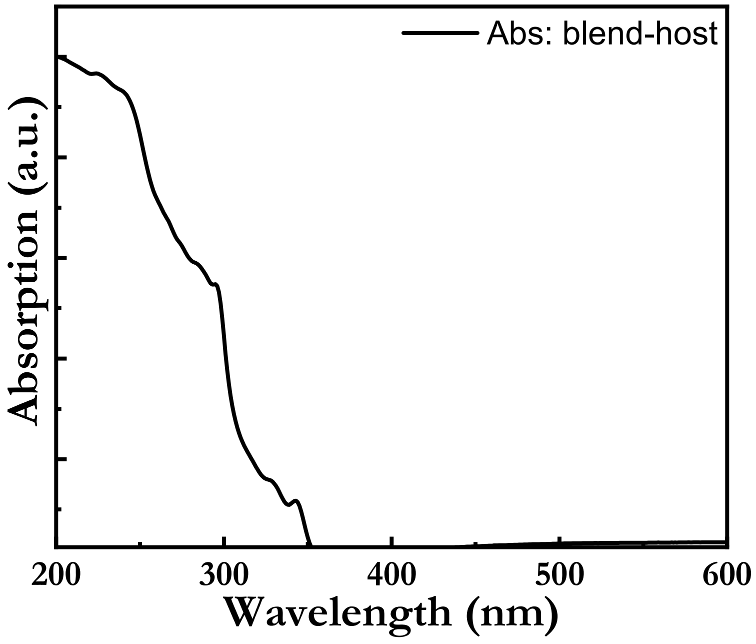

Supplementary Figure 4. Absorption of the blend-host. The absorption spectrum of a neat film of the TCTA:26DCzPPY blend-host.

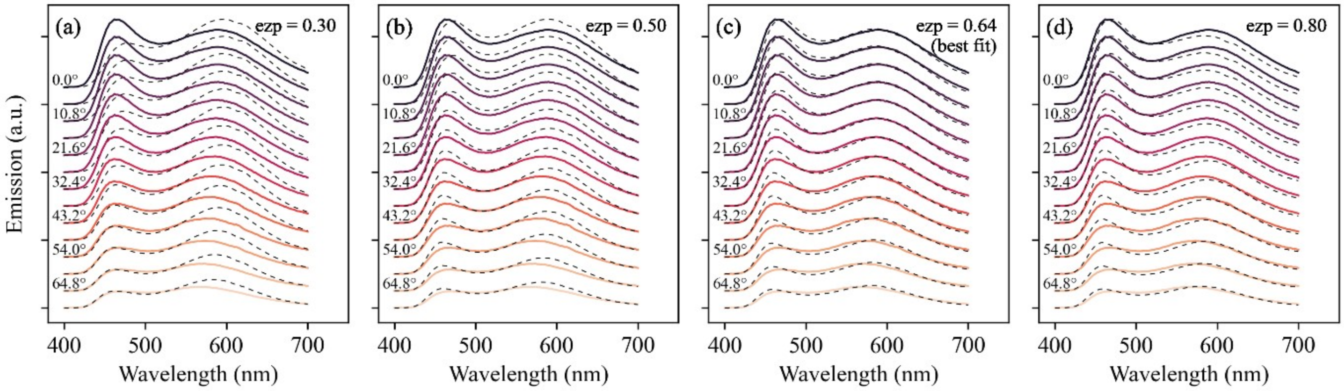

Supplementary Figure 5. Derivation of the emission zone position in the active material. The measured EL spectrum (solid lines) and the simulated EL spectrum (dashed lines) as a function of viewing angle for a white TADF-LEC equipped with a 120 nm thick active material. The EL spectra were recorded at peak luminance. The emission zone position (ezp) was systematically varied in the simulations: (a) ezp = 0.30, (b) ezp = 0.50, (c) ezp = 0.64, (d) ezp = 0.80. A value of 0 for the ezp corresponds to it being pinned at the interface with the transparent ITO anode while a value of 1 corresponds to it being located at the interface with the reflective Al cathode. The best fit of the measured emission spectra was obtained with an ezp value of 0.64, which implies that the emissive p-n junction is formed at 36 % of the active-material thickness away from the reflective Al cathode.

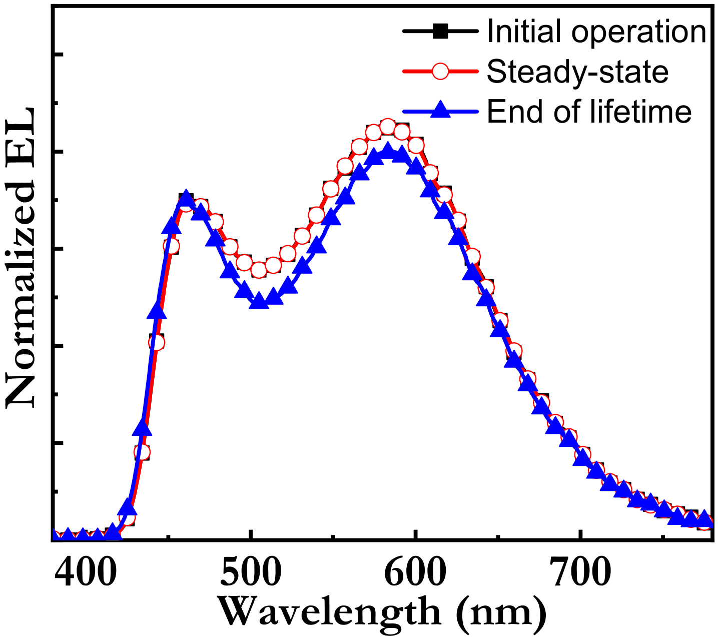

Supplementary Figure 6. **Long-term spectral stability of the white TADF-LEC.** The normalized EL spectrum of a white TADF-LEC during the initial operation, at steady-state, and close to the end of its lifetime. The EL spectrum was normalized to the intensity at λ = 465 nm. The device was driven by a constant current density of 7.7 mA/cm^2.

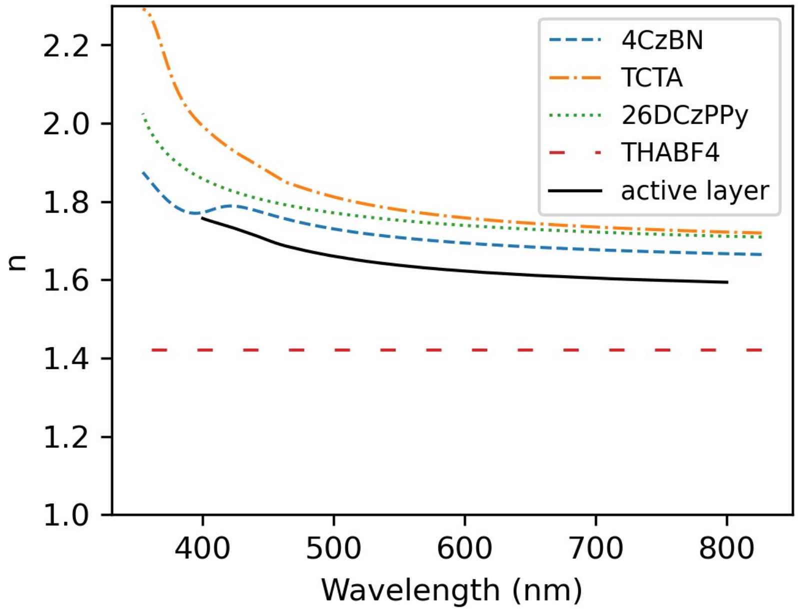

Supplementary Figure 7. **Refractive index dispersion**. The refractive index as a function of wavelength for the active layer, and its four different constituents, of the white TADF-LEC.
